# Microbiomes in Acne Vulgaris and Their Susceptibility to Antibiotics in Indonesia: A Systematic Review and Meta-Analysis

**DOI:** 10.3390/antibiotics12010145

**Published:** 2023-01-11

**Authors:** Lili Legiawati, Paulus Anthony Halim, Magna Fitriani, Hardya Gustada Hikmahrachim, Henry W. Lim

**Affiliations:** 1 Department of Dermatology and Venereology, Faculty of Medicine, Universitas Indonesia, Cipto Mangunkusumo National Central General Hospital, Jakarta 10430, Indonesia; 2 Faculty of Medicine, Universitas Indonesia, Jakarta 10430, Indonesia; 3 Department of Dermatology, Henry Ford Health, Detroit, MI 48202, USA

**Keywords:** antibiotic resistance, Indonesia, acne vulgaris, *Cutibacterium acnes*, *Staphylococcus epidermidis*, *Staphylococcus aureus*, tetracyclines, macrolides, clindamycin

## Abstract

Hot and humid countries such as Indonesia have a higher prevalence of acne vulgaris (AV). The activity of skin microbes, not only *Cutibacterium acnes*, contribute to the formation of AV. Topical and oral antibiotics are routinely prescribed to treat AV. As antimicrobial resistance rates increase globally, there are concerns about decreased efficacy. This study intends to systematically evaluate the microbiomes isolated from AV lesions and their antibiotics susceptibility in Indonesia. The data were retrieved through PubMed, EMBASE, Google Scholar, and ScienceDirect searches for articles published until July 2022 using three multiword searches. Sixteen studies published between 2001 and 2022 were identified from which the data were pooled using a random effects model. The pooled prevalence estimates demonstrated that *C. acnes*, *Staphylococcus epidermidis*, and *Staphylococcus aureus* were the three common microbes associated with AV in Indonesia. Tetracyclines had lower resistance rates compared to those of macrolides and clindamycin, with *C. acnes* showing a resistance rate that is as high as 60.1% against macrolides. *C. acnes* resistance against minocycline showed an increasing trend, whereas the resistances to doxycycline, clindamycin, and macrolides stagnated. The high resistance prevalence and trends signify a public health concern. The results of this study call for the development of antibiotic stewardship programs in Indonesia, which may lead to improved acne outcomes.

## 1. Introduction

Acne vulgaris (AV) is a chronic inflammatory disorder that primarily affects adolescents and young adults, and it can present as comedones, papules, pustules, nodules, and erythema. It is a multifactorial disease resulting from the interplay of genetics and environmental factors [1]. Increased sebum excretion, the proliferation of the pilosebaceous unit, bacterial growth, and inflammation are the four pathogenesis of AV. Antibiotics are routinely prescribed for acne patients due to their effects in suppressing the latter two mechanisms. However, with many countries reporting high rates of *Cutibacterium acnes* (or *C. acnes*, formerly *Propionibacterium acnes*) resistance [2,3], there are concerns about decreased efficacy [4]. Other microbes, such as *Staphylococcus aureus* and *Staphylococcus epidermidis*, are also involved in acne pathogenesis, and they may contribute to the development of resistance due to cross-resistance [5,6].

Various consensus-based guidelines recommend that topical and oral antibiotics are not used as monotherapies to limit resistance [7,8,9]. The current American guideline suggests that benzoyl peroxide (BPO) in conjunction with a topical retinoid and/or antibiotics is the first-line therapy for mild-to-moderate AV [10]. Oral antibiotics could be added to a combination therapy for moderate-to-severe AV. The European guideline strongly recommends oral isotretinoin for moderate-to-severe papulopustular or nodular AV, however, oral antibiotics in combination with topical treatments could also be recommended [11]. A previous review reported varying rates of antibiotic-resistant *C. acnes* in several countries, with the lowest rate of resistance being seen in Chile, and the highest one being seen in Spain [2]. The patterns of antimicrobial resistance (AMR) are heterogeneous across countries, therefore, domestic data are essential for clinical decisions related to acne management. 

Acne vulgaris is more prevalent in regions with a higher temperature and humidity [12]. In Indonesia, a tropical country, almost every adolescent and young adult has to deal with the disease. Acne vulgaris is one of the most common reasons for dermatology office visits in Indonesia [13]. Presently, cumulative resistance data of pathogens associated with acne vulgaris in Indonesia are unavailable. This lack of data can be attributed to reduced laboratory capabilities and gaps in surveillance methods and practices. In this light, a systematic review and meta-analysis (SRMA) was conducted to determine the pattern of microorganisms isolated from acne vulgaris lesions and their susceptibility to antimicrobials in Indonesia. This study aimed to provide guidance for the development of future strategies against antibiotic resistance in acne management by presenting the current resistance data of pathogens associated with acne vulgaris. In addition, the results of this study provide a high-quality evidence for clinicians in selecting antibiotics for patients with acne vulgaris.

## 2. Results

### 2.1. Literature Search

**Figure 1:**
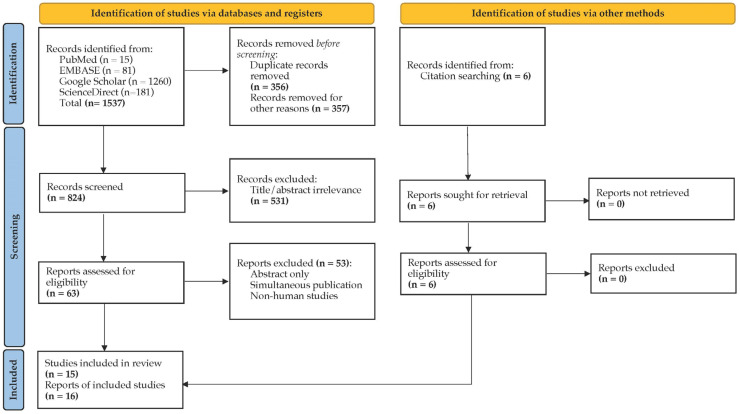
A PRISMA 2020 flow diagram of the article selection process.

The search strategy for this SRMA is represented using a Preferred Reporting Items for Systematic Reviews and Meta-analyses (PRISMA) flow diagram (Figure 1). An initial search from four databases yielded 1537 articles, of which 713 were excluded following duplicate removal and a pre-screening check. A further 531 records were excluded after manually screening the titles and abstracts, and 53 records were excluded after the manually screening of the full-text review. The article by Sari et al. [14] presented data reported in two included manuscripts [15,16], and thus, it was excluded from this study. Six additional reports were identified from the references of screened articles. Finally, 16 reports were included for the quantitative and qualitative analyses.

### 2.2. Study Characteristics and Risk of Bias

The characteristics of the included studies are summarized in Table 1. Sixteen studies were dated from 2001 to 2022. Nine studies were conducted on Java island, four studies were conducted on Sumatera island, two studies were conducted on Sulawesi, and one study was conducted on West Nusa Tenggara. Overall, 733 acne vulgaris patients were subjected to bacterial identification and antimicrobial susceptibility testing, or the former only. All of the included studies were cross-sectional and conducted from 2000 to 2020. The subjects included in the studies were primarily teenagers or adults of both sexes, and they were aged 10–39 years with mild-to-severe acne. The studies cultured specimens from inflammatory (i.e., papules, pustules, and nodules) and non-inflammatory (i.e., comedones) lesions. The most common exclusion criterion was a history of antibiotic use, both oral and topical ones. The characteristics of 11 studies that reported AMR testing are outlined in Table 2. Overall, the quality of included studies was satisfactory (Figure S1).

### 2.3. Bacterial Identification and Resistance Testing Methods

All of the studies conducted bacterial identification using cultures. In addition, two studies conducted in Makassar used polymerase chain reaction (PCR) for the identification. Anaerobic cultures were utilized in 12 studies, with seven of those studies also using aerobically cultured acne specimens. Three studies did not specify the culture methods utilized. A colorimetric system (VITEK 2 (bioMérieux, Durham, NC, USA)) was used the most for bacterial identification following the culture step, however, older methods such as Gram staining and biochemical tests were still used in some studies. Disk diffusion was the most utilized AMR testing method. Tetracycline, minocycline, doxycycline, erythromycin, clindamycin, and azithromycin were the most commonly used antibiotics in these tests.

### 2.4. Microorganisms Isolated

**Figure 2:**
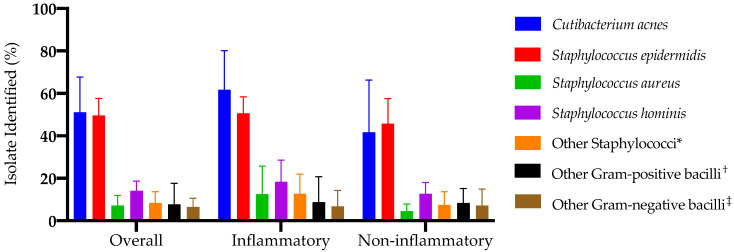
Microorganisms isolated from acne vulgaris lesions in the included studies. Subgroup analysis was performed based on acne lesion type. Error bars represent upper 95% confidence intervals. * *S. arlettae*; *S. auricularis*, *S. capitis*, *S. cohnii*, *S. haemolyticus*, *S. lugdunensis*, and *S. vitulinus*; *S. warneri* and *S. xylosus*; ^†^ *Actinomyces odontolyticus*, *Bacillus* spp., *Clostridium* spp., *Corynebacterium urealyticum*, and *Bacillus* spp.; ^‡^ *Citrobacter koseri*, *Enterobacter* spp., *Klebsiella* spp., and *Lactobacillus plantarum*; *Providencia stuartii*, *Aeromonas veronii*, and *Pseudomonas aeruginosa*.

The pattern of microorganisms isolated from AV lesions in the included studies is summarized in Figure 2. Overall, *C. acnes* and *S. epidermidis* were the two predominant bacterial species, with a pooled prevalence of 51.2% (95% CI: 34.5–67.7) and 49.6% (95% CI: 41.5–57.6), respectively. The pooled prevalence rate of *S. aureus* from AV lesions was 7.1% (95% CI: 3.4–11.9). *Staphylococcus hominis* and other staphylococci were sporadically isolated, with pooled prevalence rates of 14.1% (95% CI: 10.1–18.7) and 8.5% (95% CI: 4.3–13.7), respectively. Various other Gram-positive and Gram-negative bacilli were sparingly identified, with pooled prevalence rates of 8.4% (95% CI: 3.4–15.2) and 6.6% (95%: 3.3–10.7). Forest plots for each bacterial species or group are presented in Figures S2–S8.

There was significant heterogeneity between the studies, as indicated by *I*^2^ values of 95%, 72%, 55%, 58%, and 67% in computing the pooled prevalence rates of *C. acnes*, *S. epidermidis*, *S. aureus*, other staphylococci, and other Gram-positive bacilli, respectively. Hence, subgroup analyses were performed based on the type of acne lesion. *Cutibacterium acnes* was more commonly identified in inflammatory lesions than non-inflammatory ones were, with pooled prevalence values of 61.7% (95% CI: 41.5–80.1) and 41.7 (95% CI: 19.0–65.3), respectively. Furthermore, inflammatory lesions grew *S. aureus* more frequently compared to non-inflammatory ones, with prevalence estimates of 12.7% (95% CI: 3.5–25.7) and 4.6% (95% CI: 2.1–7.8), respectively. The presence of *S. epidermidis* and other Gram-positive and Gram-negative bacilli did not differ when they were stratified by lesion type. The data regarding the history of antibiotics use and the gender of the subjects were not reported in the majority of the included studies.

An observation of the funnel plots indicated small study effects for the estimate of the *C. acnes* prevalence, which was statistically confirmed by Egger’s test (Figure S2). A sensitivity analysis was performed by excluding studies with a small sample size (*n* < 30). Data from the remaining larger studies showed that the prevalence of *C. acnes* became lower (39.9%, 95% CI: 23.7–57.3), but the heterogeneity remained high (*I*^2^ = 95%). The funnel plots of the other prevalence estimates showed no obvious evidence of publication bias, as presented by Egger’s test for all of the estimates, except for the prevalence of other staphylococci (Figures S3–S8).

### 2.5. Antimicrobial Resistance

Eleven out of the sixteen included studies reported AMR testing. All eleven studies studied a resistance to clindamycin, doxycycline, and erythromycin, whereas resistance to azithromycin, minocycline, and tetracycline was assessed in four (36.4%), seven (63.6%), and ten (90.9%) studies, respectively. The AMR rates were pooled for the three most commonly identified pathogens, *C. acnes*, *S. epidermidis*, and *S. aureus*, against tetracycline, minocycline, doxycycline, erythromycin, clindamycin, and azithromycin (Figures S9–S11). 

A similar pattern of AMR was observed for the three pathogens (Table 3), in which the pooled resistance rates for macrolides were higher than they were for tetracyclines. The resistance rates to clindamycin were similar to those of the macrolides. Compared to all the antimicrobials studied, resistance to doxycycline was the least frequent, whereas erythromycin was the most prevalent one, with resistance being observed in approximately half of all isolates identified. In addition to the antibiotics included in Table 3, Ruchiatan et al. tested the resistance to levofloxacin (25.0%), cotrimoxazole (46.4%), and cefadroxil (14.3%) [28]. Hapsari et al. reported a moderate resistance to cotrimoxazole and chloramphenicol, but there was no resistance to levofloxacin and ciprofloxacin [25]. Sari et al. reported a high sensitivity to ciprofloxacin (89.6%) and levofloxacin (92.7%).

**Figure 3:**
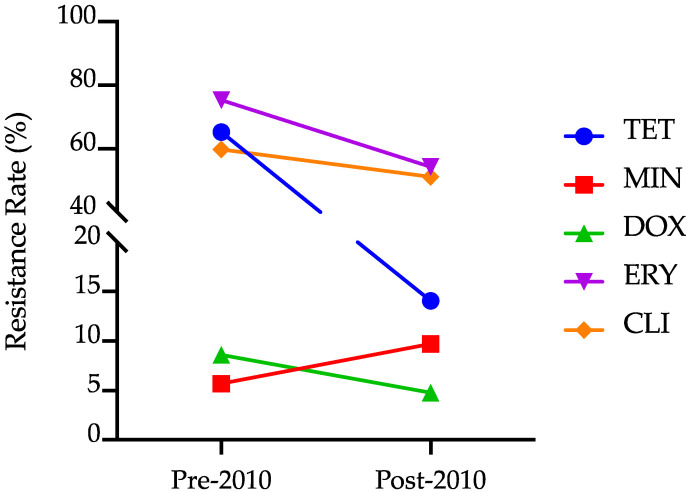
The resistance rates of *Cutibacterium acnes* against tetracycline (TET), minocycline (MNO), doxycycline (DOX), erythromycin (ERY), and clindamycin (CLI) after stratifying by study period (before and after 2010).

*Cutibacterium acnes* showed resistance to all of the antibiotics commonly used to treat acne. Among the tetracyclines, the pooled resistance rates of *C. acnes* against minocycline (9.0%, 95% CI: 3.6–16.0) and doxycycline (5.6%, 95% CI: 2.5–9.6) were lower compared to that for tetracycline (28.5%, 95% CI: 10.7–50.1). Interestingly, the *C. acnes* strains isolated from acne lesions in Indonesia showed a similar resistance for clindamycin (53.3%, 95% CI: 38.4–68.0) compared to that for macrolides (i.e., erythromycin (60.1%, 95% CI: 42.5–76.5) and azithromycin (53.6%, 95% CI: 19.5–86.0)). The pooled resistance rates for tetracycline, minocycline, erythromycin, and azithromycin showed significant heterogeneity, which prompted a subgroup analysis based on the study year. By dividing the studies into those conducted before and after 2010, the resistance for tetracycline was significantly higher in the 2000s (65.3%, 95% CI: 25.7–95.9) compared to that in the following decade (14.1%, 95% CI: 8.6–20.5). The pooled resistance rates for minocycline, doxycycline, erythromycin, and clindamycin were not statistically different between the two periods. Isolates of *Cutibacterium acnes* were increasingly resistant to minocycline, whereas the resistance to doxycycline, erythromycin, and clindamycin stagnated (Figure 3).

Similar to *C. acnes*, the isolates of *S. epidermidis* from the acne lesions in Indonesia showed high resistance rates to macrolides (i.e., erythromycin (58.5%, 95% CI: 48.7–67.1) and azithromycin (52.4, 95% CI: 41.4–63.3)) and clindamycin (54.8, 95% CI: 40.5–68.7). Among the tetracyclines, minocycline (0.0%, 95% CI: 0.0–2.4) had the least resistance against *S. epidermidis*, which was followed by doxycycline (11.9%, 95% CI: 4.3–21.9) and tetracycline (24.8, 95% CI: 13.2–38.5). The resistance rates of *S. aureus* to minocycline (0.0%, 95% CI: 0.0–13.5), doxycycline (13.2%, 95% CI: 0.0–44.9), and erythromycin (42.1%, 95% CI: 22.5–62.8) were similar to that of *S. epidermidis*. However, *S. aureus* was more susceptible to tetracycline (9.2%, 95% CI: 0.0–34.7), azithromycin (5.4%, 95% CI: 0.0–22.9), and clindamycin (21.3%, 95% CI: 0.0–67.3).

## 3. Discussion

Our systematic review and meta-analysis focused on the microbes isolated from AV lesions and their antibiotic susceptibility in Indonesia published in 2001–2022. Our pooled estimates showed that *C. acnes*, *S. aureus*, and *S. epidermidis* were the three common pathogens associated with AV, with the first two species being more commonly isolated from inflammatory lesions. This study also revealed that those pathogens were more susceptible to tetracyclines compared to macrolides and clindamycin.

In this study, the two most commonly isolated bacteria from acne lesions were *C. acnes* and *S. epidermidis*. This finding is in correlation with the results of previous studies, including studies that utilized bacterial culture [30,31,32] as well as genetic sequencing [33,34]. We found that *C. acnes* was the predominant bacterial species in inflammatory acne lesions, whereas *S. epidermidis* tends to dominate non-inflammatory lesions. Previous sequencing studies reported that *Cutibacterium* was more abundant in inflammatory acne lesions compared to non-inflammatory ones [35,36]. A recent work by Akaza et al. also demonstrated that *Cutibacterium* spp. was the main bacteria in the comedonal contents of inflammatory acne lesions [33]. In contrast, a quantitative PCR from the work by Xu et al. demonstrated that the content of *C. acnes* was statistically decreased in the inflammatory acne group [35]. Loss et al. also reported that *S. epidermidis* was more common in comedones compared to inflammatory acne lesions [36].

In inflammatory acne, the *C. acne* heat shock protein induces skin macrophages to produce numerous pro-inflammatory cytokines, such as interleukin-6 (IL-6) and IL-8. The latter one stimulates neutrophil migration, which generates oxygen-free radicals that kill microorganisms. However, excessive free radicals stimulated by C. acnes leads to free radical leakage into the extracellular area, destroying the follicular epithelium and contributing further to the development of inflammatory reactions [37]. 

*S. epidermidis* has been successfully used as a probiotic acne patch, and it has been proven to limit the growth of *C. acnes*, therefore reducing the inflammation induced by *C. acnes* [38]. *S. epidermidis* and *C. acnes* are known to cooperate in the pathogenesis of acne. Short-chain fatty acids (SCFAs) produced by *S. epidermis* and *C. acne* are present in acne lesions as antimicrobial agents that compete with each other. This process uses glycerol as a carbon source. Currently, there is no evidence suggesting the active contribution of *S. epidermidis* in acne pathogenesis [5]. The exopolysaccharide intercellular adhesin (PIA) of *S. epidermidis* forms a biofilm that protects the microorganism from the innate human immune system and supports the growth of *C. acnes* [14].

The role of *S. aureus* in acne pathogenesis is controversial. This study revealed that *S. aureus* is more commonly isolated from inflammatory acne lesions, which suggests that it might be implicated in the development of inflammatory acne. Previous works have reported that the virulence of *S. aureus* is enhanced by *C. acnes* presence, forming a denser biofilm [39,40]. However, Khorvash et al. showed that the colonization rate of *S. aureus* was similar in acne patients compared to that of healthy controls [41].

In this study, the prevalence of antibiotic resistance mainly stagnated in Indonesia. The finding correlates to a previous review by Karadag et al. [3], who reported that the prevalence of antibiotic resistance of acne has increased for many years, reaching its peak of 75% in the early 2000s. During the past decade, however, the global prevalence of antibiotic resistance in acne has decreased to 30–40% [3]. Compared to the global estimate [3,42], our study revealed that the antibiotic resistance prevalence among Indonesian acne patients was higher.

**Figure 4:**
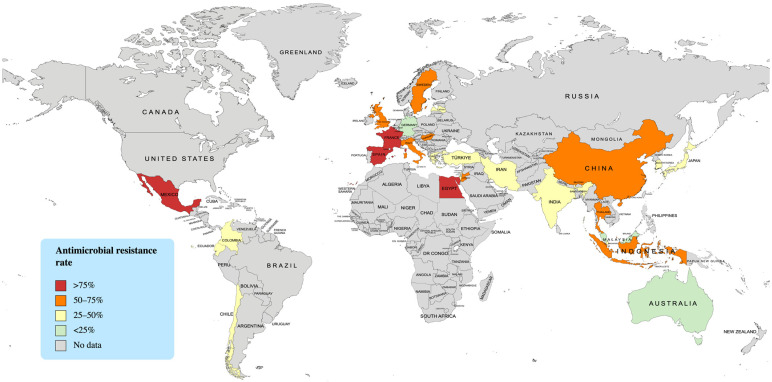
Global distribution of *Cutibacterium acnes* antimicrobial resistance. The map was generated using MapChart.net (https://mapchart.net/world.html, accessed on 10 January 2023).

The resistance of *C. acnes* to various antibiotics in different countries is summarized in Table 4 and Figure 4. Tetracyclines resistance in Indonesia is relatively low compared to those in several Asian countries (i.e., Hong Kong, Israel, and Jordan), and it is comparable to those in most European countries. However, the resistance to macrolides and clindamycin in Indonesia is among the highest globally.

In this study, erythromycin and clindamycin had the highest resistance rates. The finding is consistent with studies from other countries in Asia, Europe, and the Americas, which reported a higher rate of resistance to macrolides compared to that for tetracyclines. This finding might be explained by the fact that erythromycin and clindamycin are commonly prescribed for acne due to their efficacy and minor side effects [2]. Furthermore, antibiotic monotherapy is still prescribed for AV, with a recent study in the US reporting the occurrence of it in up to 11.7% and 25.6% of dermatologist and non-dermatologist visits, respectively [67]. Similar data in Indonesia are not yet available, however, the occurrence of antibiotic monotherapy for acne vulgaris is expected to be high owing to the low perception of AMR issues among healthcare practitioners and patients [68,69]. Interestingly, considering that doxycycline had become the preferred oral antibiotic for AV treatment in the past decade [9,70], our study still reported minimal doxycycline resistance. This might be partly explained because doxycycline is not routinely prescribed in Indonesia for indications other than AV, whereas macrolides are more commonly used for other indications [71,72]. Macrolides were also reported to be more frequently consumed by Indonesians who practice self-medication using antibiotics [73].

Another intriguing finding is that resistance of *C. acnes* to minocycline was more prevalent compared to that for doxycycline. Compared to reports from other countries, only South Korea reported the same pattern. The rationale for this observation is unclear, as minocycline is less commonly used for AV due to its side effect. This might be partially explained by the different tetracycline resistance mechanisms circulating in various countries, which may result from mutations in the 16S rRNA subunit or efflux pumps [74]. Further studies regarding the molecular basis of *C. acnes* resistance in Indonesia are warranted.

Antibiotics remain one of the most widely used therapies that are used to treat acne vulgaris. However, topical or oral antibiotics should not be used as monotherapies. One strategy to reduce antibiotic resistance is to add BPO when long-term oral antibiotics are needed [8]. Topical antibiotics are frequently combined with an antimicrobial agent (e.g., BPO) to treat mild acne. Previous works have reported the inhibitory effect of BPO on *C. acnes* biofilm formation [75,76], thus limiting the possibility of resistance development. Topical retinoids and antimicrobial agents, which are added to a course of oral antibiotics, are recommended as the first-line regimen for treating moderate-to-severe acne [9]. Antibiotics for acne are generally not prescribed for more than three or four months. According to one study, dermatologists have a higher propensity to prescribe antibiotics, two-thirds of which are for acne [77]. However, the problem we face today is that antibiotic resistance rates are increasing worldwide.

Despite there being high macrolide and lincosamide resistance rates in Indonesia, topical erythromycin and clindamycin are still commonly prescribed for AV in Indonesia [78,79]. Furthermore, self-medication with topical and oral antibiotics is widespread in Indonesia, with most pharmacies and drug stores freely dispensing antibiotics without prescription [80]. These practices might contribute to the persistently high rate of *C. acnes* resistance to macrolide and clindamycin in Indonesia over the past two decades. The stricter use and distribution of antibiotics are recommended to limit further antibiotic resistance development.

An update to the national guidelines on the treatment of AV needs to address the high rate of macrolide and lincosamide resistance reported in this work. Oral doxycycline or minocycline could be given for moderate-to-severe acne, however, the use of oral minocycline might be limited due to numerous adverse effects. Treatments with oral antibiotics should be evaluated from every 4 to 6 weeks in terms of the response and to ensure compliance. In addition, therapy for moderate–severe AV should include topical retinoic acids and/or BPO, which are applied regularly over the surface of all the acne-affected skin.

Sarecycline, a novel narrow-spectrum tetracycline, was approved by the FDA in 2018 for treating moderate-to-severe AV [81]. The new antibiotic was demonstrated to have a low propensity for resistance development [82]. Furthermore, alternative or adjuvant therapies with agents that act on the acne microbiota, such as antimicrobial peptides [83], photodynamic therapy [84], *C. acnes* bacteriophages [85], or probiotics [86] would prevent resistance. The development and application of these agents should be encouraged in the fight against AMR.

To the best of our knowledge, this study is the first to systematically review and analyze the microbiome of AV and their antimicrobial susceptibility pattern in Indonesia. To optimize the relevant studies, we included unpublished theses and contacted the authors of conference proceedings. The included studies were mostly conducted in Java, Sumatra, and the Sulawesi islands, hence, the results of our work might not represent other parts of the Indonesian archipelago.

## 4. Materials and Methods

### 4.1. Literature Search Strategy and Selection

This study was performed following the PRISMA and MOOSE guidelines [87,88]. The protocol of this study was registered on the International Prospective Register of Systematic Reviews (PROSPERO) database (registration number: CRD42022314459). Four electronic databases (PubMed, EMBASE, ScienceDirect, and Google Scholar) were queried in July 2022 to identify the relevant articles. The text headings and medical subject headings (MeSH) terms used for the search strategy included: (“acne vulgaris” OR “acne” OR “akne” OR “akne vulgaris” OR “jerawat”), (“antimicrobial” OR “antibiotics” OR “antimicrobial resistance” OR “antibiotik”), (“microbiome” OR “bacteriology” OR “bacteria” OR “*Cutibacterium*” OR “*Propionibacterium*” OR “*Staphylococcus*”), and Indonesia. Each key concept (i.e., the set of terms within the parentheses) was combined with the Boolean operator AND, except for the antimicrobial and microbiome concepts which were combined with OR. We also searched the reference lists of included studies and relevant review articles. The database searches included unpublished theses and dissertations. We contacted the authors of conference proceeding abstracts to obtain the full-length reports.

### 4.2. Inclusion and Exclusion Criteria

The studies that reported bacterial identification with or without the AMR testing of acne vulgaris lesions in Indonesia, regardless of age, severity, and the type of acne lesion, were considered eligible for inclusion. Only full-length reports in English or Indonesian language were included in the analysis. Case reports, animal, and laboratory studies were excluded.

### 4.3. Data Extraction and Quality Appraisal

All of the screened studies were retrieved and organized using EndNote 20 reference management software to remove the duplicates. Two independent reviewers (P.A.H. and M.F.) reviewed all of the publication titles and abstracts for eligibility. Any disagreements were discussed with a senior reviewer (L.L.). One author extracted data into a standardized data collection form, and another one verified the accuracy of the extracted data from the source article. The publication year, first author’s name, data collection time, study design, study location, and sample size were recorded. The type of sample of the acne lesions, the severity of the acne, the bacterial culture and identification methods, and the methods of antibiotic susceptibility tests were also recorded. The isolated bacterial species and their resistance patterns were collected. Any isolates reported as intermediately susceptible were considered to be resistant isolates.

The quality of each eligible study was assessed by two reviewers (P.A.H. and M.F.) independently using the Joanna Briggs Institute (JBI) Critical Appraisal Checklist [89]. The checklist is comprised of nine questions, in which each “Yes” answer to a question earns a study one point. Any disagreements were resolved by a discussion involving a senior author (L.L.).

### 4.4. Statistical Analysis

The meta-analysis was performed using the Metaprop function in STATA 14.0 (StataCorp LP, College Station, TX, USA). The species of microorganisms identified and their number of isolates were pooled to represent the overall pattern of the microbiota of acne vulgaris among the included studies. Graphical figures were generated using GraphPad Prism 9.0 (GraphPad Software, Inc., San Diego, CA, USA). The pooled resistance rates for each antibiotic were analyzed for *C. acnes*, *S. aureus*, and *S. epidermidis*. The resistance rate is the number of isolates of a specific organism that is resistant to a specific antibiotic divided by the total number of isolates of a specific microorganism, which was reported as a percentage. The homogeneity of the included studies was evaluated according to the Cochrane guidelines, and the variability between the studies was reflected using the *I*^2^ index. A value of zero implies true homogeneity, while values of 25%, 50%, and 75% imply low, moderate, and high heterogeneity, respectively. The sub-group and sensitivity analyses were performed to search for potential sources of heterogeneity. Publication bias was assessed by visually inspecting the funnel plots and testing for significance with Egger’s test. All of the tests were two-sided, with 0.05 being the significance level.

## 5. Conclusions

To the best of our knowledge, our study is the first to evaluate the microbiota of acne vulgaris and their antimicrobial resistance pattern in Indonesia using an SRMA methodology. The pooled estimates demonstrated that *Cutibacterium acnes*, *Staphylococcus aureus*, and *Staphylococcus epidermidis* were the three common pathogens associated with acne vulgaris in Indonesia. The first two pathogens were more commonly isolated from inflammatory acne lesions compared to non-inflammatory ones. Tetracyclines had a lower resistance rate in Indonesia compared to those of macrolides and clindamycin. In Indonesia, *C. acnes* resistance against minocycline showed an increasing trend, whereas the rate of resistance against tetracycline declined. The resistance rates against doxycycline, clindamycin, and macrolides stagnated. The results of this study call for the development of antibiotic stewardship programs in Indonesia to prevent the spread of AMR, especially in relation to AV treatment. The recommendations may include the restriction of antibiotics monotherapy, time limitations in using antibiotics treatments, and the utilization of novel treatment options.

## Figures and Tables

**Table 1:** Major characteristics of the 16 included studies in this systematic review and meta-analysis.

No	Study (Author, Year)	Ref	Study Period	Region	Sample Size	Acne Severity	Type of Lesions	Identification Method	Bacteria Identified
1	Soelistina, I. 2001	[17]	2000	Surabaya	67	ND	Pustule	ANC	CA
2	Barira, S. 2006	[18]	from 4/2005 to 9/2005	Jakarta	50	Moderate to severe	Comedone	ANC	CA, SE, AGPB, AGNB, cocci, AHC, AD, BF
3	Syahrial, M.A. 2009	[19]	from 12/2008 to 8/2009	Medan	43	Moderate to severe	Comedone	ANC	CA, SE, cocci, BF, AHC
4	Sylvia, L. 2010	[20]	from 8/2010 to 11/2010	Padang	33	ND	Comedone and pustule	AEC, ANC	CA, SE, SA, KS, ES, coliform,
5	Anasyifa, H. 2016	[21]	4/2016	Jakarta	10	Moderate to severe	ND	AEC	CA
6	Hindritiani, R. 2017	[22]	2014	Bandung	50	Mild to severe	Comedone, pustule, and skin swabbing	ANC	CA
7	Iryani, F. 2018	[23]	from 5/2016 to 6/2016	Makassar	45	Mild to severe	Comedone, pustule, papule, and nodule	Culture (unspecified), PCR	CA, SE, SA, BS, ES
8	Sitohang, I.B.S. 2019	[13]	from 12/2015 to 1/2016	Jakarta	93	Mild to severe	Comedone	AEC, ANC	CA, SE, SA, SH, KP, SHa. AV, CB, CD, CG
9	Asditya, A. 2019	[24]	2018	Surabaya	40	Moderate to severe	Pustule	ANC	CA, CU
10	Hapsari, Y. 2019	[25]	10/2018	Mataram	43	Moderate to severe	ND	Culture (unspecified)	SE, SA, BC, BSu, PS, AeV
11	Tabri, F. 2019	[26]	from 7/2017 to 8/2017	Makassar	43	Mild to severe	Comedone	Culture (unspecified), PCR	CA, SE, SH, SW, SX, SA, SAu, SHa, SC, LP
12	Fadilla, Y. 2019	[27]	from 1/2018 to 2/2018	Bandung	30	Mild to moderate	Pustule	AEC, ANC	CA, SE, SH, AO, SC, SAu, SL, PA, CK, PAn
13	Ruchiatan, K. 2020	[28]	from 1/2019 to 2/2019	Bandung	30	Mild to severe	Comedone	AEC, ANC	CA, SE, SH, SC, SHa, SW, PAn, KP, EA
14	Jusuf N.K. 2020	[15]	from 11/2019 to 12/2019	Medan	40	Mild to severe	Comedone and pustule	AEC, ANC	CA, SE, SH, SA, SHa, LM, ML, KV, SV, SCo, SAr, DN
15	Hermawan, M. 2021	[29]	ND	Jakarta	36	Mild to severe	Comedone, pustule, papule, and nodule	AEC, ANC	CA, SE, SA, others
16	Sari, L. 2022	[16]	from 12/2019 to 1/2020	Medan	40	Mild to severe	Comedone and pustule	AEC, ANC	CA, SE, SH, SA, SHa, LM, ML, KV, SV, SCo, SAr, DN

ND, no data; AEC, aerobic culture; ANC, anaerobic culture; PCR, polymerase chain reaction; AD, aerobic diphtheroids; AGNB, aerobic Gram-negative bacilli; AGPB, aerobic Gram-positive bacilli; AHC, alpha-hemolytic streptococci; AeV, *Aeromonas veronii*; AO, *Actinomyces odontolyticus*; AV, *Atopobium vaginae*; BC, *Bacillus cereus*; BF, *Bacteroides fragilis*; BS, *Bacillus* sp.; BSu, *Bacillus subtilis*; CA, *Cutibacterium acnes*; CB, *Clostridium bifermentans*; CD, *Clostridium difficile*; CG, *Clostridium* group; CK, *Citrobacter koseri*; CU, *Corynebacterium urealyticum*; DN, *Dermacoccus nishinomiyaensis*; EA, *Enterobacter aerogenes*; ES, *Enterobacter* sp.; KP, *Klebsiella pneumoniae*; KS, *Klebsiella* sp.; LP, *Lactobacillus plantarum*; KV, *Kocuria varians*; LM, *Leuconostoc mesenteroides*; ML, *Micrococcus luteus*; PA, *Pseudomonas aeruginosa*; PAn, *Peptostreptococcus anaerobius*; PS, *Providencia stuartii*; SAr, *S. arlettae*; SA, *S. aureus*; SAu, *S. auricularis*; SC, *S. capitis*; SCo, *S. cohnii*; SE, *S. epidermidis*; SH, *S. hominis*; SHa, *S. haemolyticus*; SL, *S. lugdunensis*; SV, *S. vitulinus*; SW, *S. warneri*; SX, *S. xylosus*.

**Table 2:** Characteristics of included studies reporting antimicrobial susceptibility tests.

No	Study (Author, Year)	Ref	Study Year	Region	AST	Tested Bacterial Species	Tested Antibiotics
1	Soelistina, I. 2001	[17]	2000	Surabaya	DD	CA	CLI, DOX, ERY, SXT, TET
2	Barira, S. 2006	[18]	2005	Jakarta	DD	CA	CLI, DOX, ERY, MNO, TET
3	Syahrial, M.A. 2009	[19]	2009	Medan	DD	CA	CLI, DOX, ERY, MNO, TET
4	Anasyifa, H. 2016	[20]	2016	Jakarta	DD	CA	CLI, DOX, ERY, TET
5	Hindritiani, R. 2017	[21]	2014	Bandung	DD	CA	CLI, DOX, ERY, MNO, TET
6	Sitohang, I.B.S. 2019	[22]	2016	Jakarta	E-test	CA, SA, SE	CLI, DOX, ERY, MNO, TET
7	Asditya, A. 2019	[24]	2018	Surabaya	DD	CA	AZI, CLI, DOX, ERY
8	Hapsari, Y. 2019	[25]	2018	Mataram	DD	SA, SE	AMX, AZI, CHL, CIP, CLI, DOX, ERY, LVX, TET, SXT
9	Fadilla, Y. 2019	[27]	2018	Bandung	DD	CA, SE, SH, AO, SC, SAu, SL, PA, CK, PAn	AZI, CFR, CLI, DOX, ERY, LVX, MNO, TET, SXT
10	Ruchiatan, K. 2020	[28]	2019	Bandung	DD	CA, SE, SH, SC, SHa, SW, PA, KP, EA	AZI, CFR, CLI, DOX, ERY, LVX, MNO, TET, SXT
11	Sari, L. 2022	[16]	2020	Medan	DD	CA, SE, SH, SA, SHa, LM, ML, KV, SV, SCo, SAr, DN	AZI, CIP, CLI, DOX, ERY, LVX, MNO, TET

AST, antimicrobial susceptibility testing; DD, disk diffusion; AO, *Actinomyces odontolyticus*, CA, *Cutibacterium acnes*; CK, *Citrobacter koseri*; DN, *Dermacoccus nishinomiyaensis*; EA, *Enterobacter aerogenes*; KP, *Klebsiella pneumoniae*; KV, *Kocuria varians*; LM, *Leuconostoc mesenteroides*; ML, *Micrococcus luteus*; PA, *Pseudomonas aeruginosa*; PAn, *Peptostreptococcus anaerobius*; SAr, *Staphylococcus arlettae*; SA, *Staphylococcus aureus*; SAu, *Staphylococcus auricularis*; SC, *Staphylococcus capitis*; SCo, *Staphylococcus cohnii*; SE, *Staphylococcus epidermidis*; SH, *Staphylococcus hominis*; SHa, *Staphylococcus haemolyticus*; SL, *Staphylococcus lugdunensis*; SV, *Staphylococcus vitulinus*; SW, *Staphylococcus warneri*; AMX, amoxicillin; AZI, azithromycin; CFR, cefadroxil; CHL, chloramphenicol; CIP, ciprofloxacin; CLI, clindamycin; DOX, doxycycline; ERY, erythromycin; LVX, levofloxacin; MNO, minocycline; TET, tetracycline; SXT, sulfamethoxazole and trimethoprim.

**Table 3:** Pooled antimicrobial resistance rates for pathogens associated with acne in Indonesia.

Antibiotics	No. of Studies	No. of Isolates	Resistance (%) [95% CIs]	*I*^2^ (%)	*p*-Value
***Cutibacterium acnes***					
**Tetracyclines**					
Tetracycline (TET)	9	221	28.5 [10.7–50.1]	90	<0.01
Minocycline (MNO)	7	179	9.0 [3.6–16.0]	41	0.10
Doxycycline (DOX)	10	258	5.6 [2.5–9.6]	14	0.32
**Macrolides**					
Erythromycin (ERY)	10	258	60.1 [42.5–76.5]	87	<0.01
Azithromycin (AZI)	4	108	53.6 [19.5–86.0]	93	<0.01
**Lincosamide**					
Clindamycin (CLI)	10	258	53.3 [38.4–68.0]	82	<0.01
***Staphylococcus epidermidis***					
**Tetracyclines**					
Tetracycline (TET)	5	132	24.8 [13.2–38.5]	60	0.04
Minocycline (MNO)	4	111	0.0 [0.0–2.4]	9	0.35
Doxycycline (DOX)	5	132	11.9 [4.3–21.9]	49	0.10
**Macrolides**					
Erythromycin (ERY)	5	132	58.5 [49.7–67.1]	0	0.59
Azithromycin (AZI)	4	86	52.4 [41.4–63.3]	0	0.53
**Lincosamide**					
Clindamycin (CLI)	5	132	54.8 [40.5–68.7]	58	0.05
***Staphylococcus aureus***					
**Tetracyclines**					
Tetracycline (TET)	3	26	9.2 [0.0–34.7]	52	0.12
Minocycline (MNO)	2	14	0.0 [0.0–13.5]	N/A	N/A
Doxycycline (DOX)	3	26	13.2 [0.0–44.9]	64	0.06
**Macrolides**					
Erythromycin (ERY)	3	26	42.1 [22.5–62.8]	0	0.63
Azithromycin (AZI)	2	19	5.4 [0.0–22.9]	N/A	N/A
**Lincosamide**					
Clindamycin (CLI)	3	26	21.3 [0.0–67.3]	80	0.01

N/A, not applicable.

**Table 4:** Resistance rates (%) of *C. acnes* to various antibiotics in different countries.

Location	Year	TET	MNO	DOX	ERY	AZI	CLI	Any AB
Indonesia (this study)	2010–2022	14	10	5	54	54	51	≥54
**Southeast Asia**								
Malaysia [43]	2012	2	0	6	8	ND	15	15
Singapore [44]	2019	6	2	9	27	ND	27	29
Thailand [45]	2017	1	ND	0	64	ND	63	≥64
**Asia**								
China [46]	2019	3	ND	1	58	59	56	≥59
Hong Kong [47]	2013	16	16	16	21	ND	54	55
India [48]	2020	7	0	2	31	ND	12	≥31
Iran [49]	2011	7	ND	2	16	2	43	≥43
Israel [50]	2020	9	11	19	25	ND	17	31
Japan [51]	2019	ND	0	3	50	ND	43	≥43
Jordan [52]	2020	36	3	37	73	ND	59	≥73
South Korea [32]	2012	3	10	7	30	ND	27	37
**Europe**								
France [53]	2010	10	ND	10	75	ND	ND	≥75
Germany [54]	2021	0	ND	ND	15	ND	4	≥15
Greece [55]	2014	ND	0	1	32	ND	29	≥32
Hungary [56]	2003	0	ND	ND	47	ND	45	52
Italy [57]	2006	2	1	ND	50	ND	41	57
Latvia [58]	2021	ND	ND	ND	30	ND	21	≥30
Spain [56]	2003	5	ND	ND	91	ND	91	94
Sweden [56]	2003	14	ND	ND	45	ND	57	58
Turkey [59]	2021	4	ND	5	30	35	23	≥35
UK [60]	2018	20	ND	ND	77	ND	77	82
**Others**								
Australia [61]	2012	<9	<9	<9	<6	ND	<6	11
Colombia [62]	2013	<10	ND	<10	35	ND	15	≥35
Chile [63]	2013	0	ND	0	13	ND	8	≥26
Ecuador [64]	2018	10	3	ND	30	ND	11	≥30
Egypt [65]	2013	26	ND	16	91	9	72	≥91
Mexico [66]	2010	14	0	20	46	82	36	≥82

AB, antibiotics; ND, no data; TET, tetracycline; MNO, minocycline; DOX, doxycycline; ERY, erythromycin; AZI, azithromycin; CLI, clindamycin.

## Data Availability

All data relevant to this review are included in the text, references, and supplementary files.

## Supplementary Materials

The following supporting information can be downloaded at: https://www.mdpi.com/article/10.3390/antibiotics12010145/s1, Figure S1: Summary of quality assessment for the sixteen included studies; Figure S2: Forest and funnel plots representing the pooled prevalence of *C. acnes* from acne lesions in Indonesia; Figure S3: Forest and funnel plots representing the pooled prevalence of *S. epidermidis* from acne lesions in Indonesia; Figure S4: Forest and funnel plots representing the pooled prevalence of *S. aureus* from acne lesions in Indonesia; Figure S5: Forest and funnel plots representing the pooled prevalence of *S. hominis* from acne lesions in Indonesia; Figure S6: Forest and funnel plots representing the pooled prevalence of other staphylococci from acne lesions in Indonesia; Figure S7: Forest and funnel plots representing the pooled prevalence of other Gram-positive bacilli from acne lesions in Indonesia; Figure S8: Forest and funnel plots representing the pooled prevalence of other Gram-negative bacilli from acne lesions in Indonesia; Figure S9: Forest plot representing the pooled resistance rates of *Cutibacterium acnes* against various antibiotics; Figure S10: Forest plot representing the pooled resistance rates of *Staphylococcus epidermidis* against various antibiotics; Figure S11: Forest plot representing the pooled resistance rates of *Staphylococcus aureus* against various antibiotics.
