# Supplementary material for: Microbiomes in Acne Vulgaris and Their Susceptibility to Antibiotics in Indonesia: A Systematic Review and Meta-Analysis
Source: Antibiotics (Basel). 2023 Jan 11;12(1):145. doi: 10.3390/antibiotics12010145 (PMC9854683; doi:10.3390/antibiotics12010145)
Supplement: Supplementary file 1 [file antibiotics-12-00145-s001.zip › antibiotics-2136107-supplementary.pdf]

Supplementary Figures

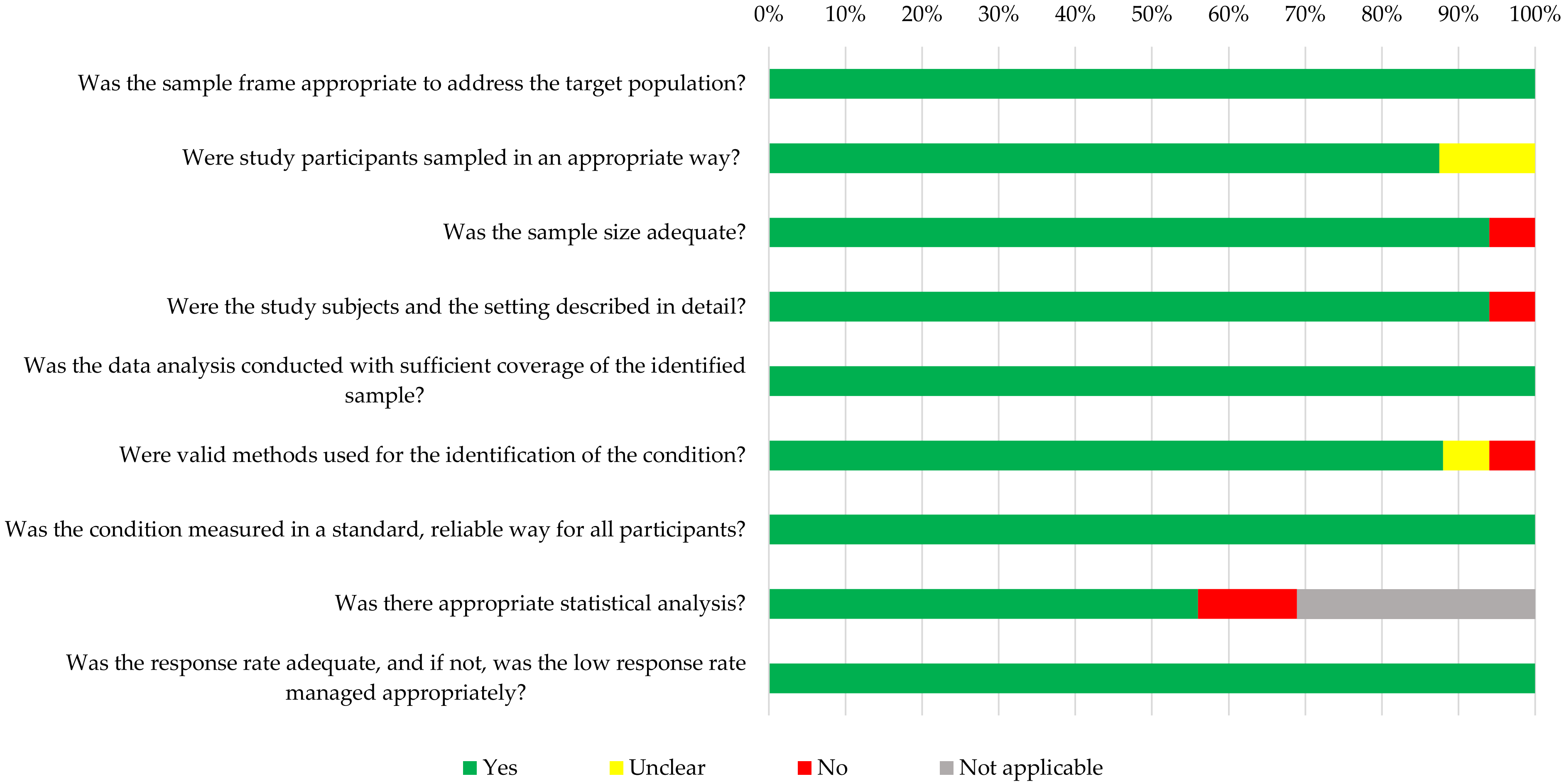

Figure S1. Summary of quality assessment for the sixteen included studies.

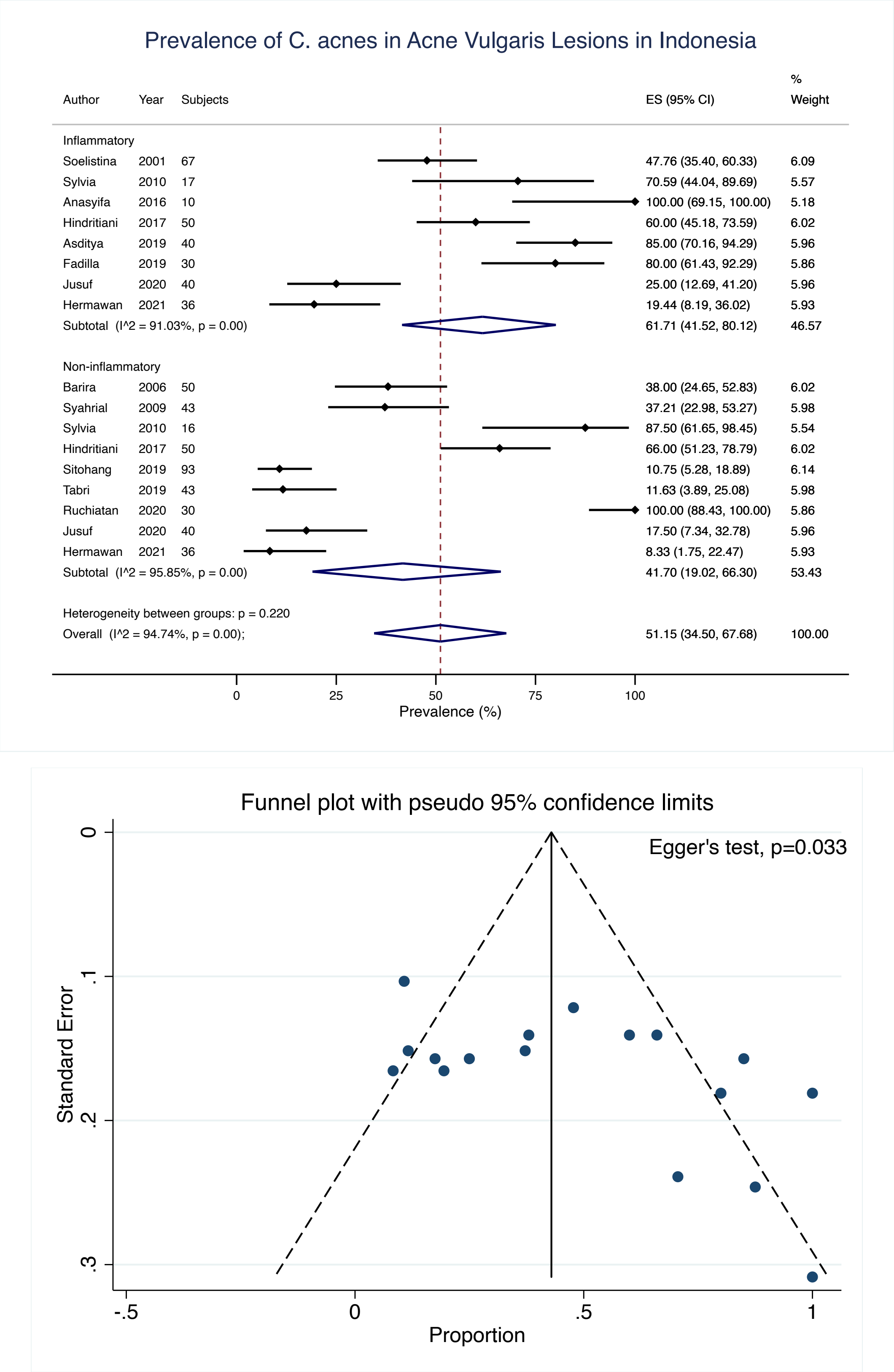

Figure S2. Forest and funnel plots representing the pooled prevalence of *C. acnes* from acne lesions in Indonesia. The pooled estimates were computed from 14 studies using the random-effects model (top panel), with subgroup according to the type of acne lesion (inflammatory or non-inflammatory lesion). The distribution of effect estimates is shown in a funnel plot (bottom panel). Figures were generated using STATA software.

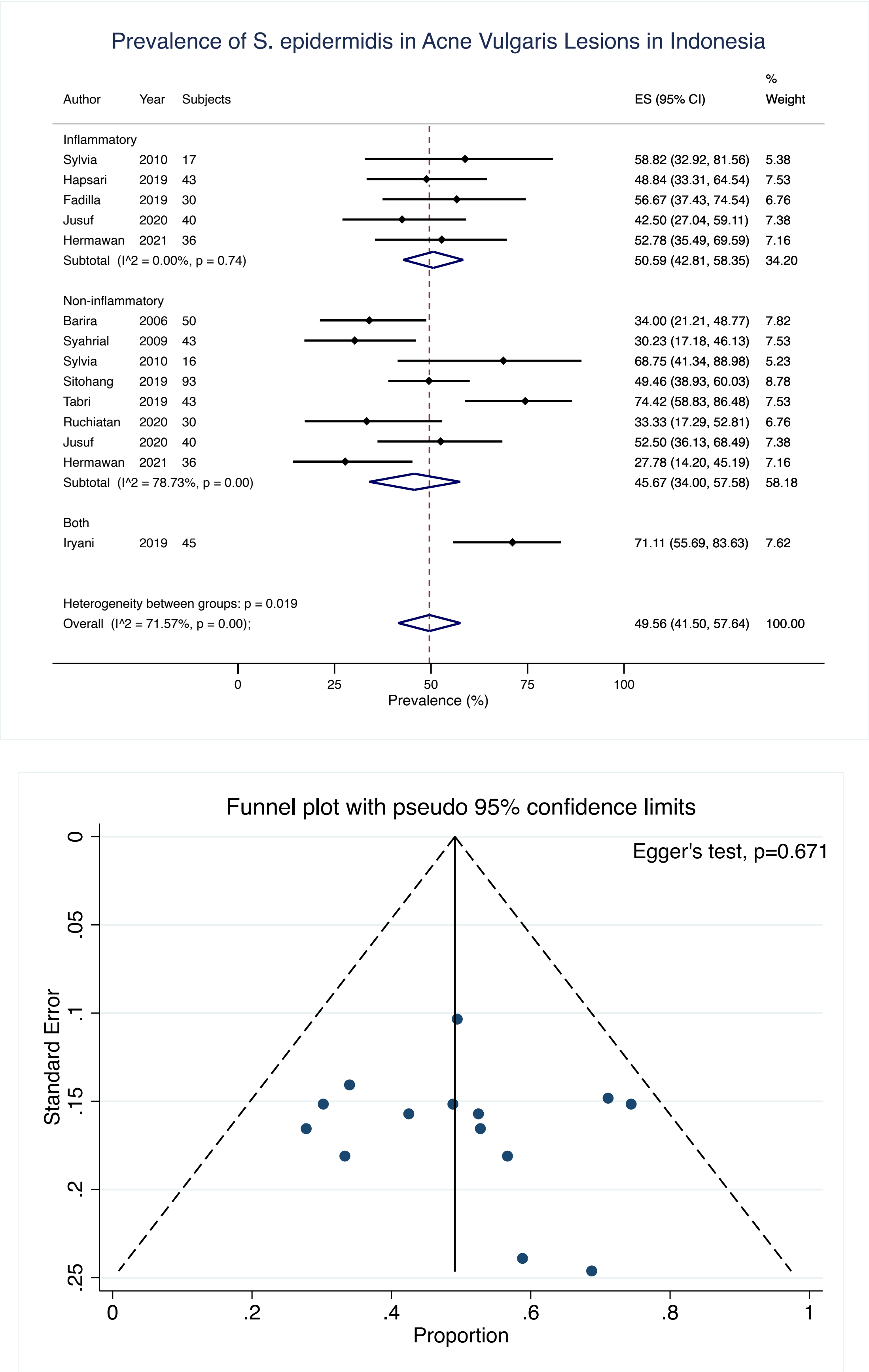

Figure S3. Forest and funnel plots representing the pooled prevalence of *S. epidermidis* from acne lesions in Indonesia. The pooled estimates were computed from 11 studies using the random-effects model (top panel), with subgroup according to the type of acne lesion (inflammatory or non-inflammatory lesion). The distribution of effect estimates is shown in a funnel plot (bottom panel). Figures were generated using STATA software.

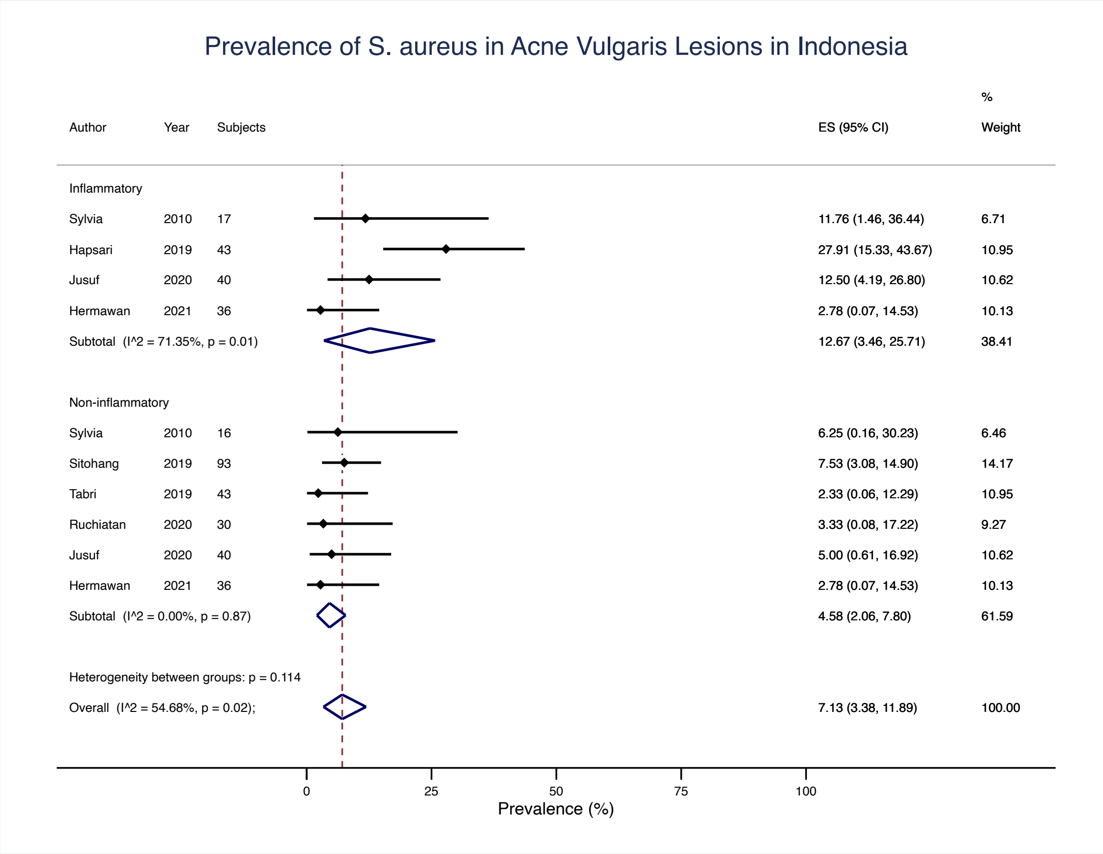

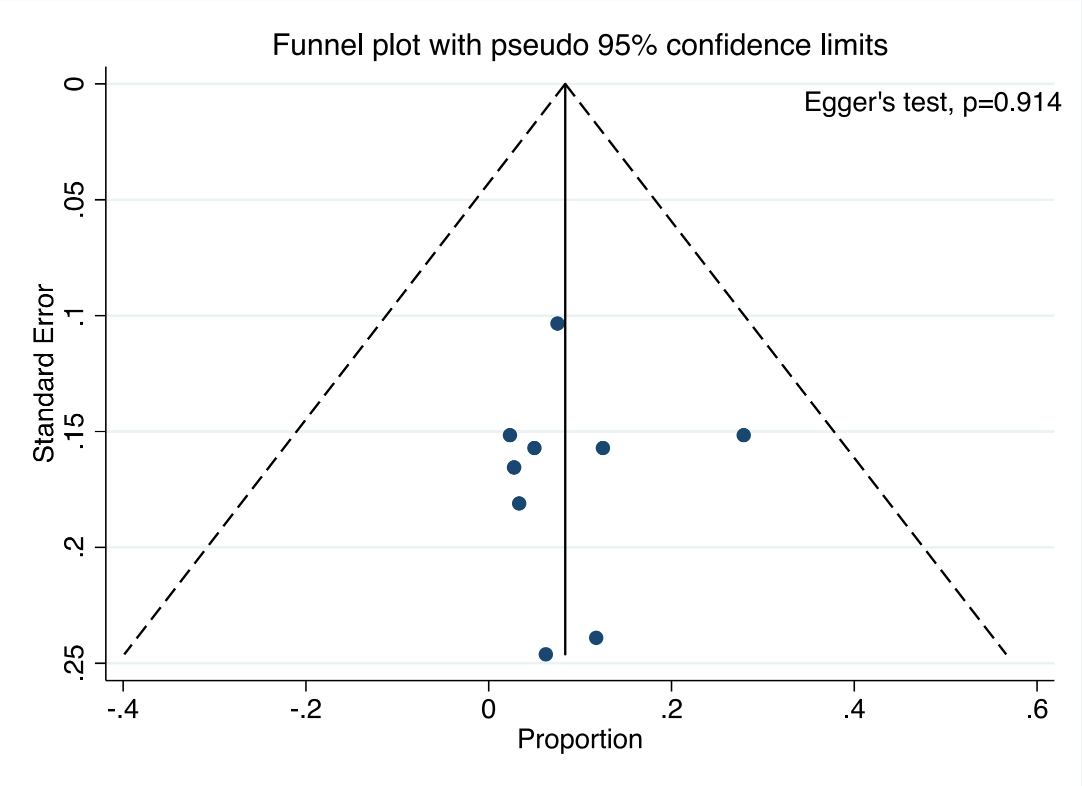

Figure S4. Forest and funnel plots representing the pooled prevalence of *S. aureus* from acne lesions in Indonesia. The pooled estimates were computed from seven studies using the random-effects model (top panel), with subgroup according to the type of acne lesion (inflammatory or non-inflammatory lesion). The distribution of effect estimates is shown in a funnel plot (bottom panel). Figures were generated using STATA software.

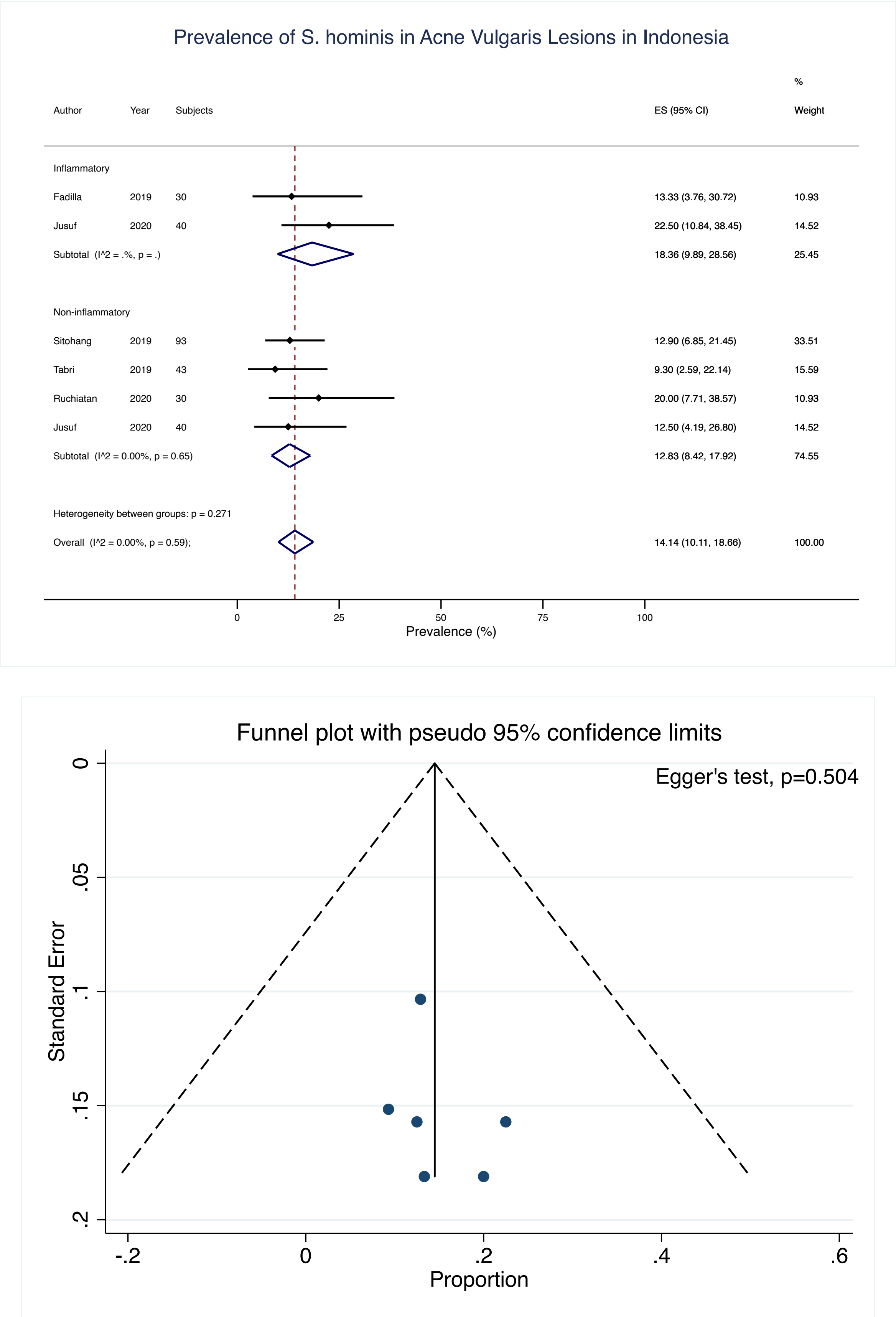

Figure S5. Forest and funnel plots representing the pooled prevalence of *S. hominis* from acne lesions in Indonesia. The pooled estimates were computed from five studies using the random-effects model (top panel), with subgroup according to the type of acne lesion (inflammatory or non-inflammatory lesion). The distribution of effect estimates is shown in a funnel plot (bottom panel). Figures were generated using STATA software.

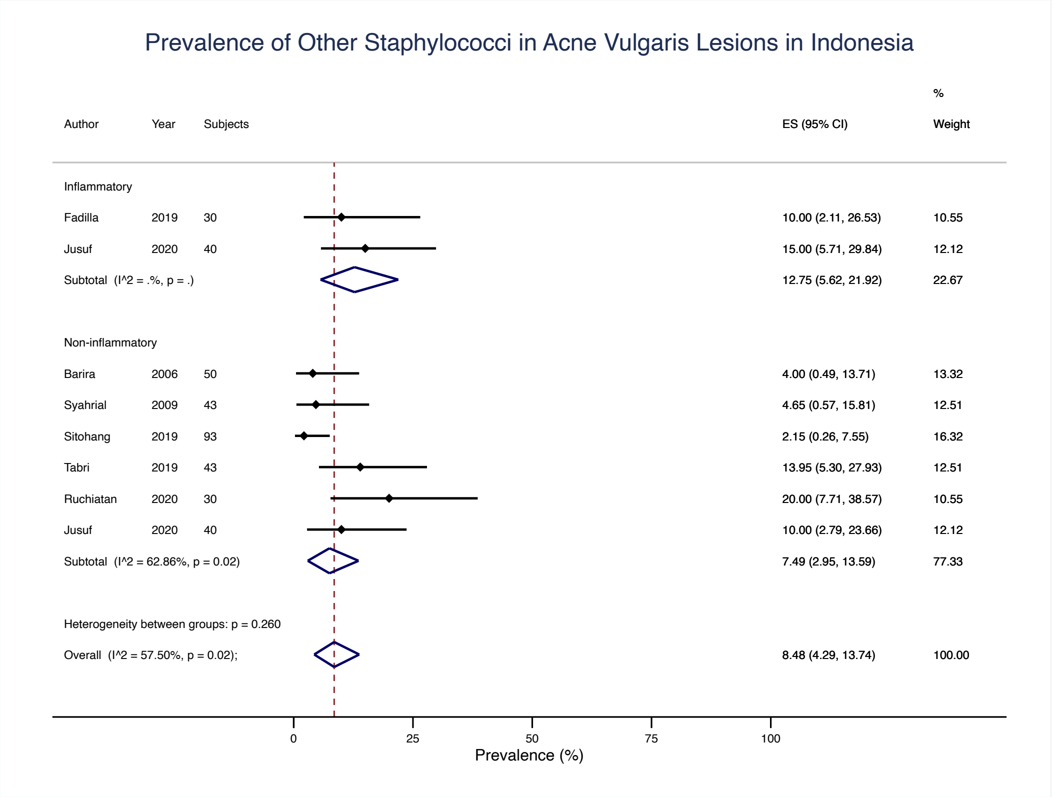

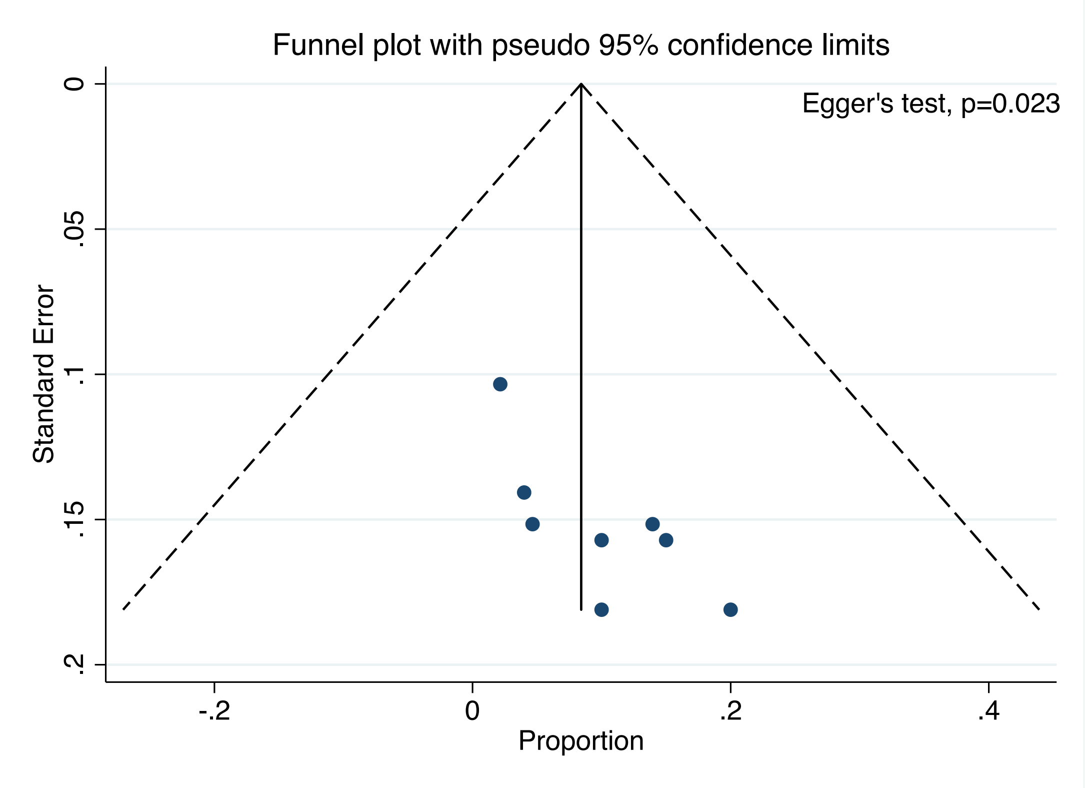

Figure S6. Forest and funnel plots representing the pooled prevalence of other staphylococci from acne lesions in Indonesia. The pooled estimates were computed from seven studies using the random-effects model (top panel), with subgroup according to the type of acne lesion (inflammatory or non-inflammatory lesion). The distribution of effect estimates is shown in a funnel plot (bottom panel). Figures were generated using STATA software.

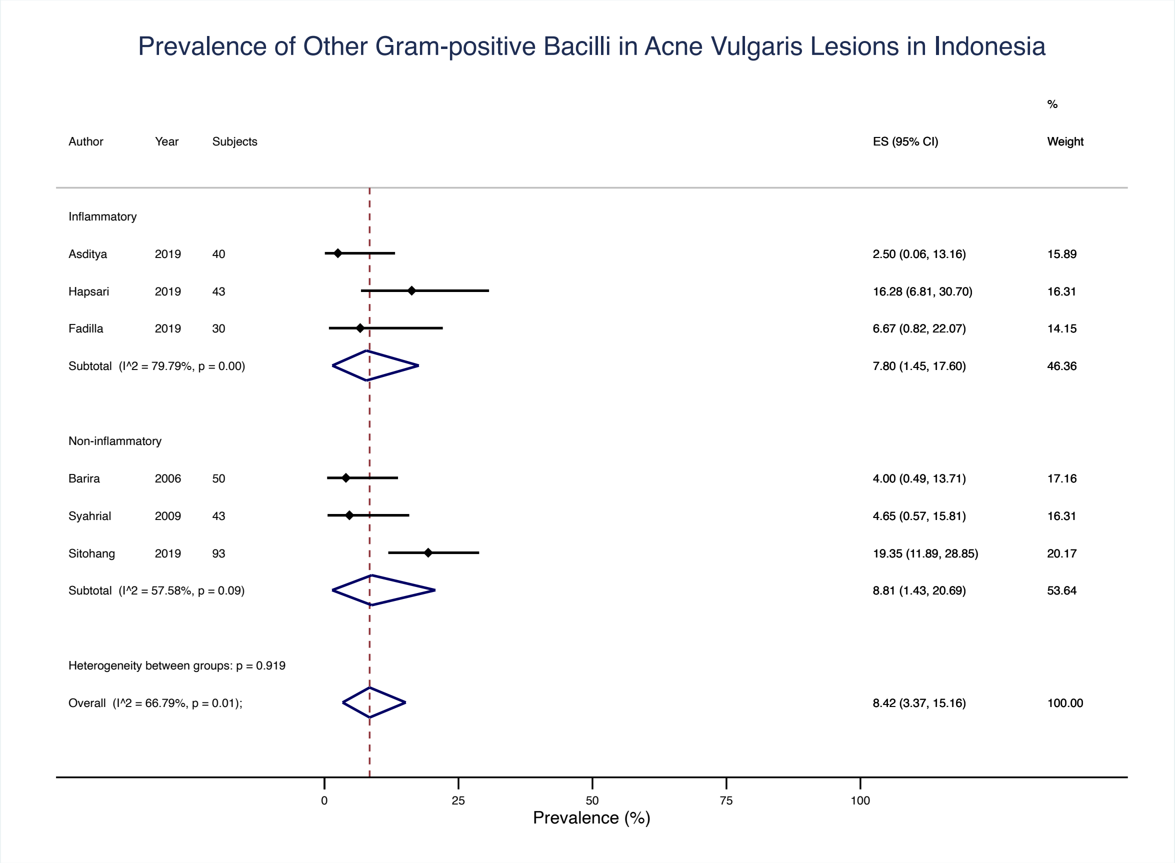

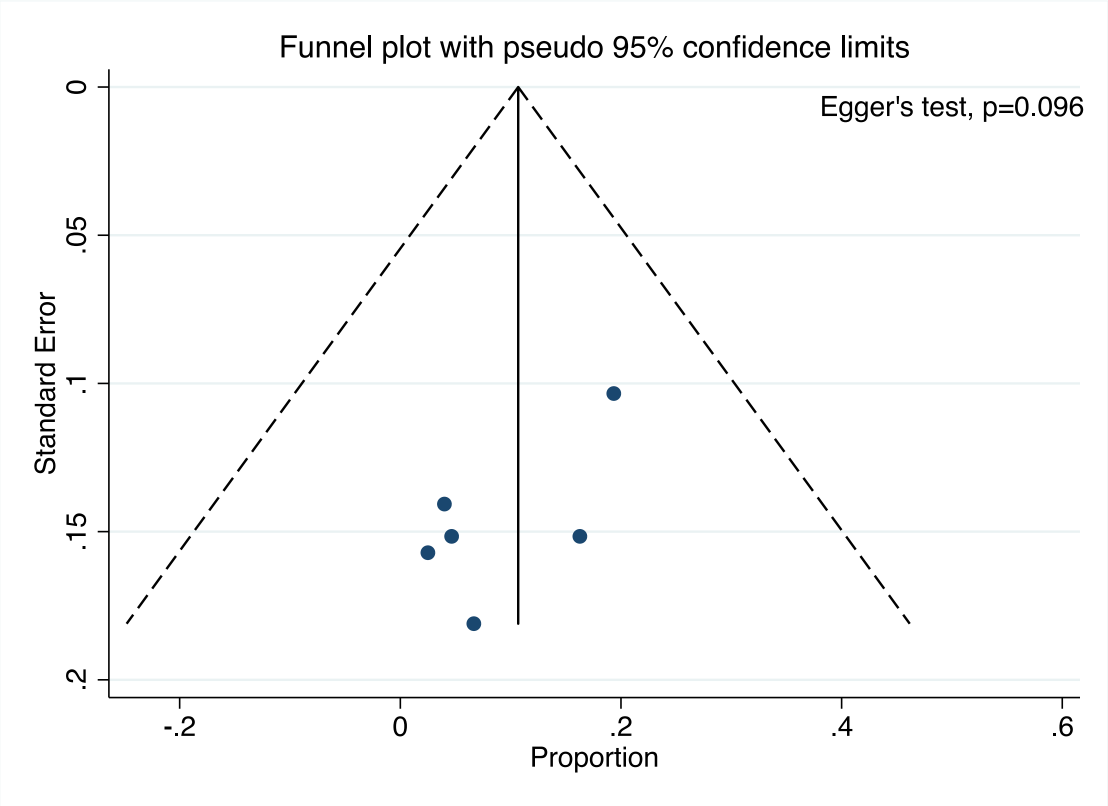

Figure S7. Forest and funnel plots representing the pooled prevalence of Gram-positive bacilli from acne lesions in Indonesia. The pooled estimates were computed from six studies using the random-effects model (top panel), with subgroup according to the type of acne lesion (inflammatory or non-inflammatory lesion). The distribution of effect estimates is shown in a funnel plot (bottom panel). Figures were generated using STATA software.

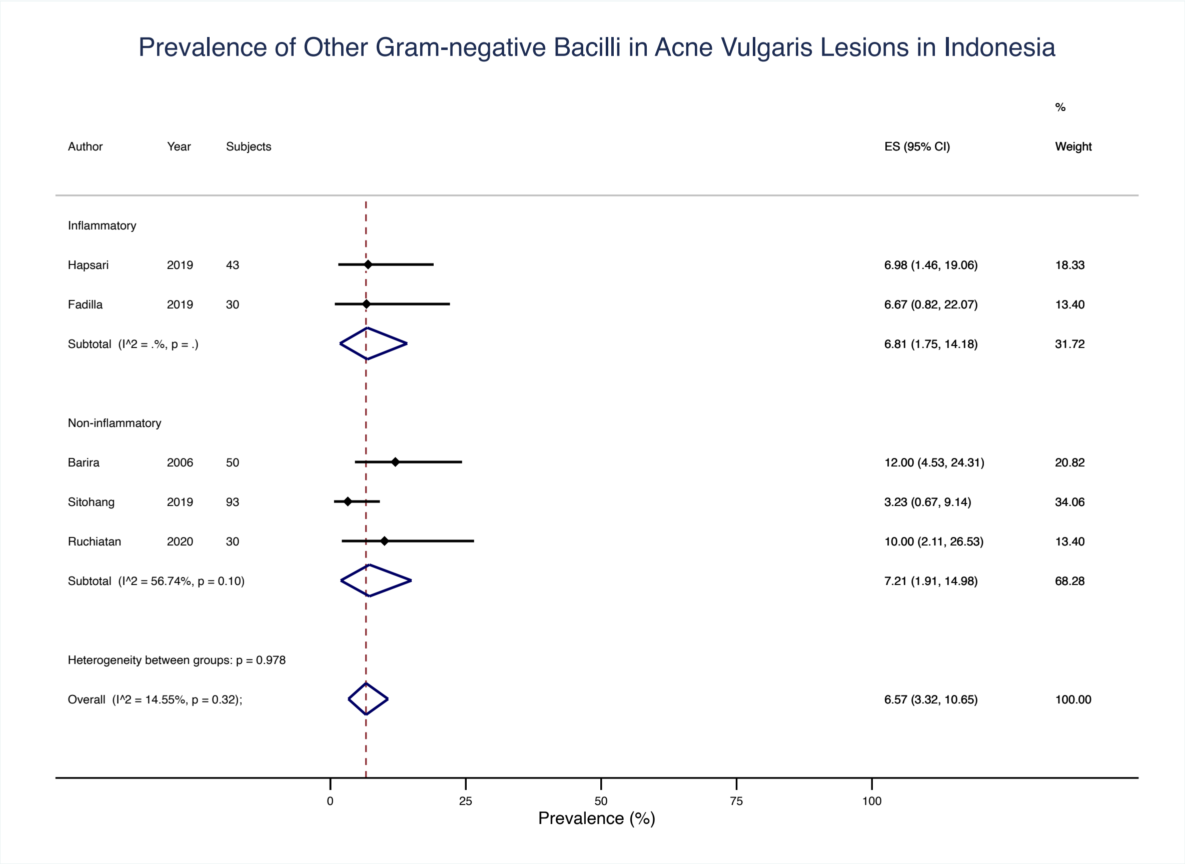

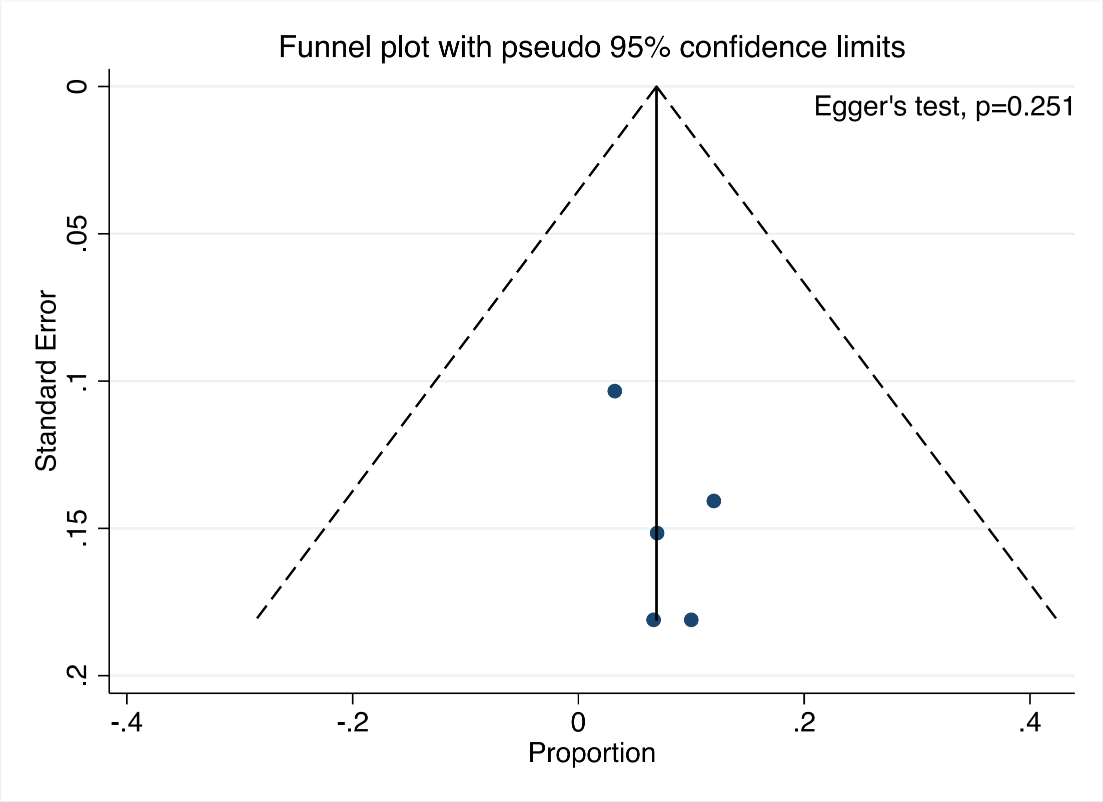

Figure S8. Forest and funnel plots representing the pooled prevalence of other Gram-negative bacilli from acne lesions in Indonesia. The pooled estimates were computed from five studies using the random-effects model (top panel), with subgroup according to the type of acne lesion (inflammatory or non-inflammatory lesion). The distribution of effect estimates is shown in a funnel plot (bottom panel). Figures were generated using STATA software.

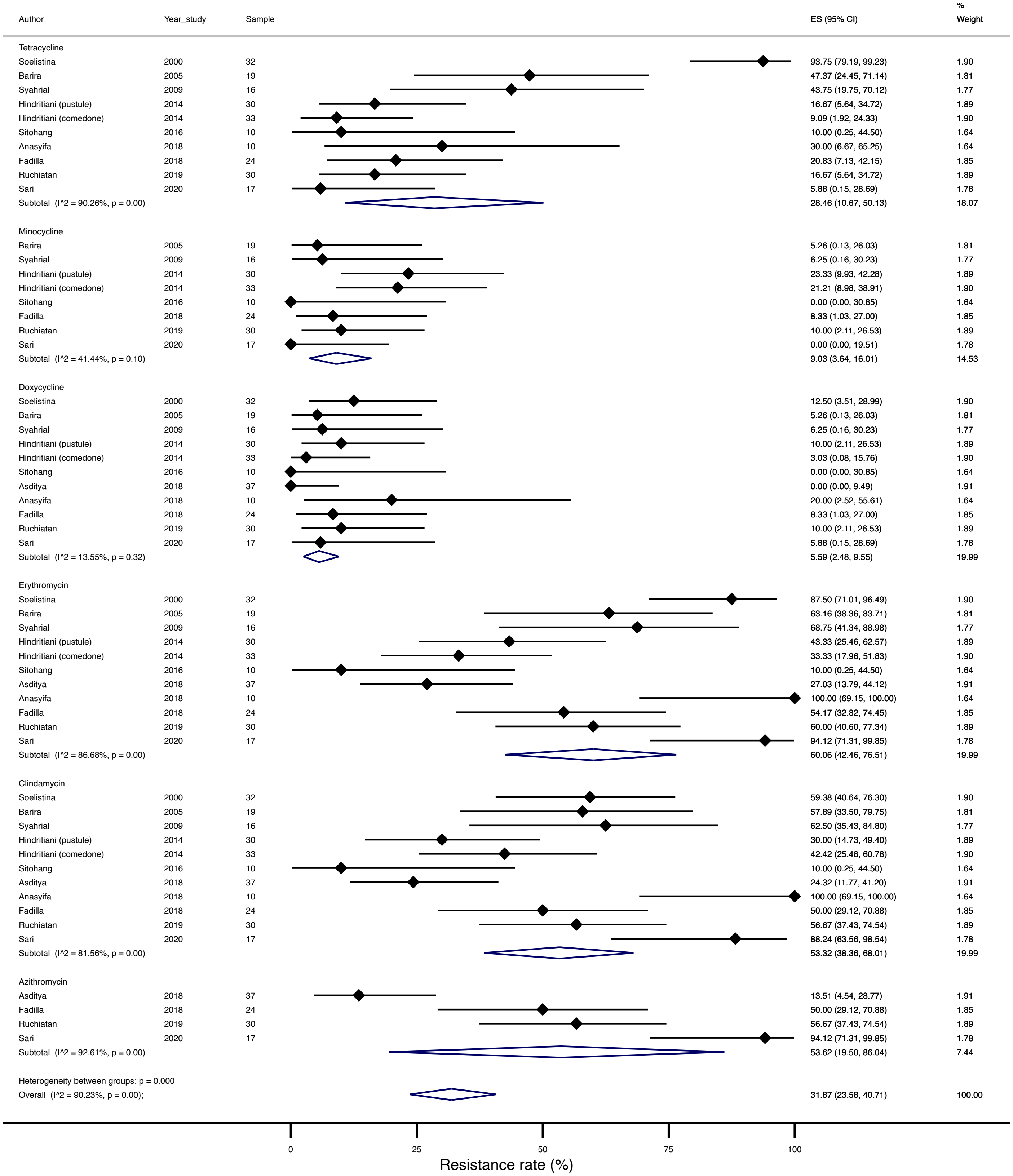

Figure S9. Forest plot representing the pooled resistance rates of *Cutibacterium acnes* against various antibiotics. Plot was generated using STATA software.

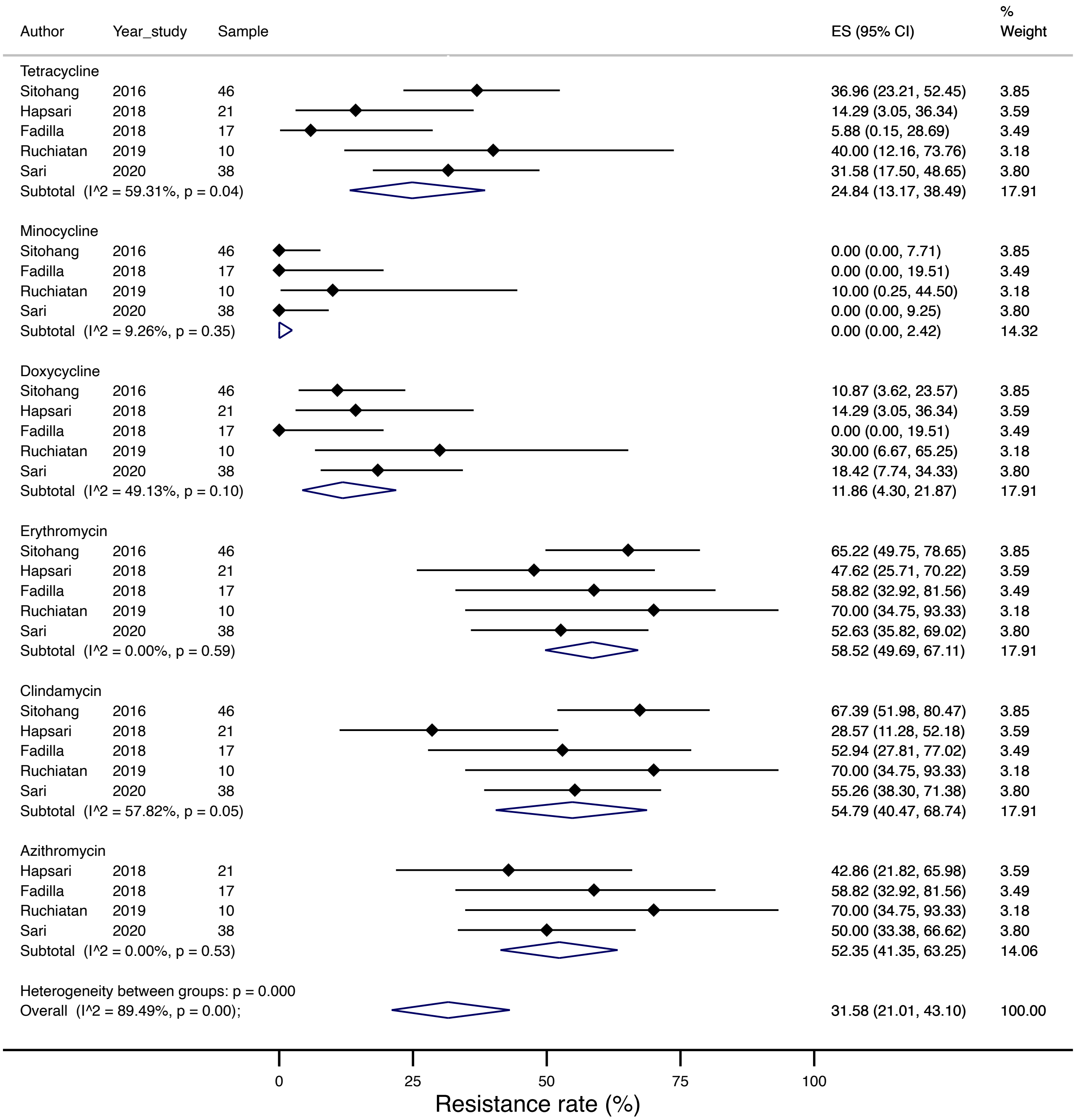

Figure S10. Forest plot representing the pooled resistance rates of *Staphylococcus epidermidis* against various antibiotics. Plot was generated using STATA software.

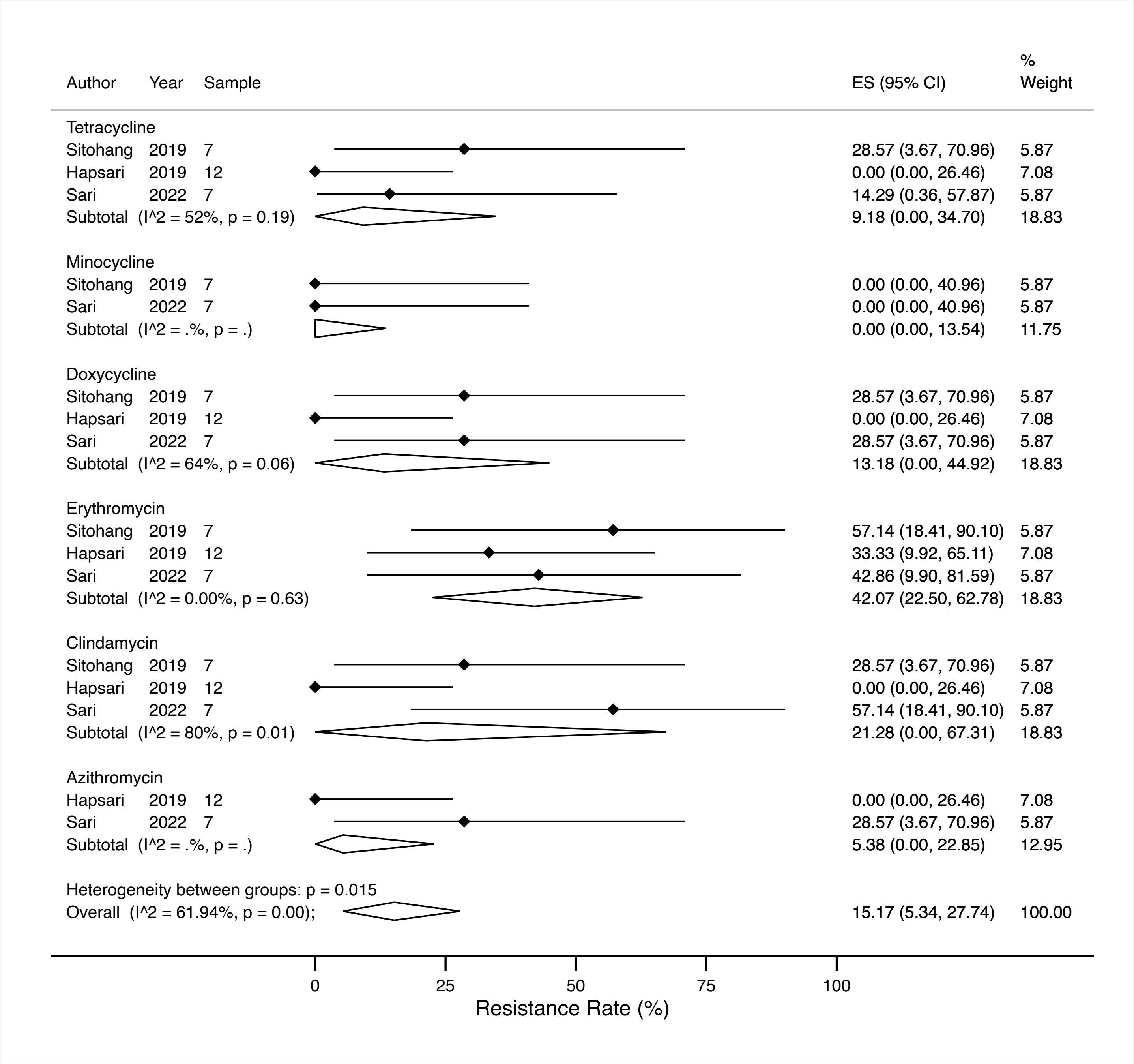

Figure S11. Forest plot representing the pooled resistance rates of *Staphylococcus aureus* against various antibiotics. Plot was generated using STATA software.
